# A pilot study on the effect of cognitive training on BDNF serum levels in individuals with Parkinson’s disease

**DOI:** 10.3389/fnhum.2015.00130

**Published:** 2015-03-16

**Authors:** Francesco Angelucci, Antonella Peppe, Giovanni A. Carlesimo, Francesca Serafini, Silvia Zabberoni, Francesco Barban, Jacob Shofany, Carlo Caltagirone, Alberto Costa

**Affiliations:** ^1^Department of Clinical and Behavioural Neurology, IRCCS Santa Lucia FoundationRome, Italy,; ^2^Department of Systemic Medicine, University of Rome Tor VergataRome, Italy

**Keywords:** Parkinson’s disease, cognitive deficits, cognitive rehabilitation, BDNF, serum levels

## Abstract

Parkinson’s disease (PD) patients, besides motor dysfunctions, may also display mild cognitive deficits (MCI) which increase with disease progression. The neurotrophin brain-derived neurotrophic factor (BDNF) plays a role in the survival of dopaminergic neurons and in the regulation of synaptic connectivity. Moreover, the brain and peripheral level of this protein may be significantly reduced in PD patients. These data suggest that a cognitive rehabilitation protocol aimed at restoring cognitive deficits in PD patients may also involve changes in this neurotrophin. Thus, in this pilot study we evaluated the effect of a cognitive rehabilitation protocol focused on the training of executive functioning and measured BDNF serum levels in a group of PD patients with mild cognitive impairment, as compared to the effect of a placebo treatment (*n* = 7/8 group). The results showed that PD patients undergoing the cognitive rehabilitation, besides improving their cognitive performance as measured with the Zoo Map Test, also displayed increased serum BDNF levels as compared to the placebo group. These findings suggest that BDNF serum levels may represent a biomarker of the effects of cognitive rehabilitation in PD patients affected by MCI. However, the functional significance of this increase in PD as well as other neuropathological conditions remains to be determined.

## Introduction

Several studies have shown that patients with Parkinson’s disease (PD), besides having motor dysfunctions, may also display mild cognitive deficits in the early stage of disease which increase with disease progression (Green et al., 2002; Janvin et al., 2003; Muslimovic et al., 2005). In particular, clinical and experimental findings consistently demonstrated that, in respect to healthy subjects, PD patients exhibit poorer performance on tests tapping selected components of executive functions, such as shifting and planning (Cools, 2006; Cools and D’Esposito, 2011; MacDonald and Monchi, 2011), working memory (Cools, 2006; Cools and D’Esposito, 2011), and free recall mechanisms in the content of episodic memory (Costa et al., 2014a).

The role of dopamine system in cognitive dysfunction in PD has been increasingly documented in the last years by studies on the short-term effect of dopaminergic medication. Indeed, levodopa is converted to dopamine presynaptically with subsequent effects post-synaptically, where it binds to both D1 class receptors (including D1 and D5) and D2 class receptors (including D2, D3, and D4). Dopamine agonists on the other hand act directly on the post-synaptic system. The commonly used non-ergot dopamine agonists, such as pramipexole and ropinirol, have high affinity only for D2 class receptors (Brusa et al., 2003; Moustafa et al., 2013), where pramipexole has a higher affinity for D3 receptors and ropinirol for D2 receptors (Beaulieu and Gainetdinov, 2011). Thus, there may be fundamental differences in the functional effects of different dopaminergic drugs. At this regard, some data on healthy subjects and PD patients suggest that phasic D2 activity would be critical for allowing the flexible modification of mental representations (cognitive flexibility) whereas tonic D1 activity could sustain the ability to retain stable representations in the face of incoming information (Cohen et al., 2002; Frank, 2005; Costa et al., 2009, 2014b; Cools and D’Esposito, 2011). Coherently with this view, the hypothesis was advanced that, in the early stages of PD, dopamine efficacy on cognitive operations might be related to the regional distribution of dopamine receptors dysfunctioning. Indeed, dopamine depletion early affects the striatal regions that are rich of D2 receptors and that are highly involved in cognitive flexibility processes (Camps et al., 1990; Yeterian and Pandya, 1991; Agid et al., 1993; MacDonald and Monchi, 2011).

However, beyond the specific molecule used, dopamine administration/withdrawal was found to both improve and worsen cognitive performance of individuals with PD (see Cools, 2006 for a review). These contrasting data have been also interpreted in the context of the pattern of dopamine depletion that in PD primarily affects nigro-dorsal striatum pathways, and the dopamine projections to dorsal prefrontal cortex (highly involved in cognitive flexibility operations; Cools and D’Esposito, 2011), while the ventral tegmental regions projecting to more ventral parts of the caudate nucleus and to prefrontal and limbic regions, particularly involved in reversal learning operations, are affected later in the disease course (Yeterian and Pandya, 1991; Agid et al., 1993). In this view, on one side, dopamine replacement may restore or improve the cognitive functions related to dorsal striatal activity (e.g., shifting abilities), while, on the other side, dopamine supplementation may overdose dopamine circuitries that include the ventral striatum and ventral prefrontal cortex areas, that are relatively less affected by dopamine depletion, causing an impairment in related cognitive functions (inverted-U-shaped dopamine action; Gothham et al., 1988; Jahanshahi et al., 2010; Cools and D’Esposito, 2011).

A recent focus has been posed on the rehabilitation of cognitive deficits in individuals with PD. Although this field of research is still at the beginning, encouraging data suggest that cognitive intervention may be useful to ameliorate some aspects of executive functioning (Calleo et al., 2012; Hindle et al., 2013). In particular, Mohlman et al. (2011) found, in PD patients, a significant generalized improvement after a working memory training on different executive measures, as assessed by the Behavioral Assessment of the Dysexecutive Syndrome battery. Sammer et al. (2006) reported that a training focused on various cognitive functions including planning, working memory and strategic control, significantly improved PD patients’ performance on set-shifting and planning measures. A more recent study also document that the administration of a complex rehabilitative training that included also planning, working memory and problem solving, significantly improved PD patients’ working memory performance (Petrelli et al., 2014). Other findings also suggest that cognitive training may produce significant changes in cerebral activity of these patients (Belleville et al., 2011; Nombela et al., 2011). However, at present the mechanism of action and the biological correlates of cognitive rehabilitation in these patients are not known.

The neurotrophin brain-derived neurotrophic factor (BDNF) plays a relevant role both in promoting the survival of striatal dopaminergic neurons and in the regulation of synaptic connectivity (Gómez-Palacio-Schjetnan and Escobar, 2013). BDNF has been widely investigated in PD animal models and humans. In humans it was shown that the brain and peripheral level of this protein may be significantly reduced in PD patients as compared to healthy subjects (Scalzo et al., 2010) and that antiparkinsonian drug treatment may increase these levels (Gyárfás et al., 2010). Data from PD animal models also evidenced that BDNF may have a protective role on DA neurons. In particular, it has been demonstrated that BDNF protects DA neurons *in vitro* from the neurotoxic effects of 1-methyl-4-phenylpyridinium (MPP+) and 6-hydroxydopamine (Galpern et al., 1996) and that, prior to striatal MPP+ infusions, the implantation of fibroblasts capable of secreting transgenic human BDNF close to the substantia nigra of rats counteract the death of DA neurons (Frim et al., 1994). In addition, intrastriatal injection of BDNF prior to unilateral 6-hydroxydopamine lesioning prevents neuronal death in the substantia nigra and decreases the apomorphine-induced rotation (a measure of asymmetrical dopaminergic function; Shults et al., 1995).

Altogether these data indicate that BDNF is not only required for the survival of dopaminergic neurons but can also influence their activity in these brain regions. Thus, since PD patients have reduced peripheral and central levels of this neurotrophin, the disturbance in executive functioning may be, at least in part, explained by the negative effect of decreased BDNF availability on dopamine pathways linked to these functions (Savitz et al., 2006). After all, the role of BDNF in cognition is well defined. At this regard, it has been recently demonstrated that the reduction of activity-dependent BDNF expression in mutant mice (BDNF-KIV mice) significantly impairs spatial memory reversal and contextual memory extinction, two executive functions that require intact hippocampal-prefrontal cortex circuitry (Sakata et al., 2013). In addition, human studies on functional BDNF polymorphisms have evidenced an association between the presence of BDNF allele variants and deficits in executive functioning (Erickson et al., 2008; Koven and Carr, 2013), set-shifting tasks in particular (Gajewski et al., 2011) together with changes in cortical morphology (Pezawas et al., 2004; Bath and Lee, 2006). The mechanism by which BDNF influences cognitive flexibility or other cognitive processes is still not clear. Nonetheless, the BDNF involvement in survival of striatal dopaminergic neurons and in the regulation of synaptic connectivity suggests that this protein may constitute one of the biological correlates of cognitive rehabilitation.

Supporting this notion, it is known that BDNF serum levels in PD patients might also change after other types of rehabilitations such as intensive motor training (Frazzitta et al., 2014). These effects have been also confirmed in PD animal models (Tuon et al., 2012; Real et al., 2013) but also to other neuropathological conditions such as stroke (Mang et al., 2013), Alzheimer’s (Dao et al., 2013) and Huntington’s (Pang et al., 2006) diseases, and spinal cord injury (Macias et al., 2009). Furthermore, in non-PD subjects, it has been demonstrated that physical exercise not only improves physical functioning but also cognitive functions and BDNF peripheral levels in aged non-pathological subjects (Vaughan et al., 2014) and ameliorates depressive symptoms (Pereira et al., 2013). Regarding the effect of cognitive rehabilitation on BDNF levels, there are data showing that schizophrenic patients undergoing to a neuroplasticity-based computerized cognitive training (10 weeks) showed a significant increase in serum BDNF compared with carefully matched control subjects who engaged in 50 h of enjoyable computer games (Vinogradov et al., 2009). Moreover, this increase in BDNF correlated with improved quality of life suggesting that serum BDNF levels may serve as a peripheral biomarker for the specific effects of the cognitive training (Vinogradov et al., 2009). Despite these data, whether BDNF may increase in other forms of cognitive remediation or possibly in response to any successful behavioral (or pharmacologic) cognitive intervention is still not known.

The fact that PD patients may also display cognitive deficits and that these functions may be dependent on BDNF activity suggest that a cognitive rehabilitation protocol aimed at improving these specific cognitive functions may also involve modification of this neurotrophin. To test this hypothesis, in this study we investigated whether a cognitive rehabilitation protocol focused on the training of executive functioning is effective in producing cognitive improvements and possibly BDNF serum changes in a group of PD patients with mild cognitive impairment, as compared to the effect of a placebo treatment. In particular, the assumption that cognitive flexibility may be precociously weakened in PD patients, likely as a result of an imbalance of dopamine activity within key regions of frontal-striatal networks (i.e., caudate nucleus and prefrontal cortex; Cools and D’Esposito, 2011), makes this cognitive process an interesting target to investigate, in PD, both the effect of cognitive trainings and the possible neurobiological modifications related to BDNF activity. Accordingly, the training we here implemented was structured to specifically potentiate set-shifting, that is the ability to flexibly access to different mental representations/processes and responses according to the environmental demands. Indeed, set-shifting is retained to be one of the basic components of the executive system whose integrity would allow the implementation of more complex cognitive functions such as problem solving and planning (Miyake et al., 2000; Miyake and Friedman, 2012). At this regard, an association between flexibility and planning weakness has been suggested in PD patients (Kliegel et al., 2011; Dirnberger and Jahanshahi, 2013). Accordingly, in order to investigate the overall effect of the cognitive training we used as outcome measure the Zoo Map Test (ZMT) that taps planning in a complex situation requiring cognitive flexibility at a great extent.

## Materials and Methods

### Patients

Fifteen right-handed individuals with idiopathic PD participated to the study after giving their written informed consent. The study was approved by the Ethic Committee of the Santa Lucia Foundation. Idiopathic PD was defined according to the United Kingdom Parkinson’s Disease Society brain bank criteria (Hughes et al., 1992). In a double-blind randomized study, PD patients were assigned to two groups of treatment: experimental or placebo. Seven patients were randomly assigned to the experimental arm and eight to the placebo arm.

Inclusion criteria included the presence of a mild cognitive impairment according to criteria of Litvan et al. (2012). Specifically, patients included should show a performance below 1.5 SD from the normal population on one neuropsychological test tapping executive functioning and on another test investigating one of the following functions: working memory/attention, visual-spatial abilities, episodic memory, and language (see below for details on the neuropsychological test battery used). Neuropsychiatric, neuroradiological (CT or MR), and laboratory examinations were executed to exclude major psychiatric disorders, neurological conditions other than PD, vascular brain lesions and major systemic or metabolic diseases potentially affecting cognitive status.

The Clinical Dementia Rating Scale, the Activity and Instrumental Activity of Daily Living (Lawton and Brody, 1969) and the Pill questionnaire (Dubois et al., 2007) were administered to exclude significant changes in routine activities management. The Beck Depression Inventory (Beck et al., 1961; Visser et al., 2009) and the Apathy Evaluation Scale – Self version (Marin et al., 1991; Leentjens et al., 2008) were also administered to assess the severity of depression and apathy, respectively. At the time of assessment, all PD patients were being treated with levodopa and/or dopamine agonists (ropinirole, pramipexole, and rotigotine). Levodopa equivalent, clinical and sociodemographic characteristics of the two PD groups are reported in **Table 1**. The specific medications taken by each patient during the study are reported in **Table 2**. The dopamine medication was maintained constant during the study.

**Table 1:** **Clinical and sociodemographic characteristics of the PD patients included in the study**.

Demographic and clinical features	Experimental group	Placebo group	*F*-values	*p*-values
Age	67.6 (10.4)	71.9 (6.3)	0.96	>0.30
Years of education	11.7 (5.6)	10.6 (3.9)	0.19	>0.60
MMSE	28.3 (1.5)	28.1 (1.9)	0.03	>0.80
Beck Depression Inventory	7.3 (3.8)	8.9 (5.9)	0.37	>0.50
Apathy Evaluation Scale	33.6 (5.4)	32.0 (9.8)	0.14	>0.70
Pill Questionnaire	2.4 (0.9)	2.9 (0.9)	0.77	>0.39
ADL	4.7 (1.6)	5.7 (0.5)	3.07	>0.10
IADL	7.0 (1.8)	7.1 (0.9)	0.03	>0.80
Disease duration	5.7 (2.8)	7.9 (6.3)	0.69	>0.40
Daily levodopa equivalents	727 (319)	732 (338)	0.01	>0.90
UPDRS T0	27.1 (13.6)	24.1 (7.1)	0.29	>0.50

*Data are expressed as mean ± standard deviation, mean (SD); MMSE, Mini Mental State Examination; UPDRS, Unified Parkinson’s Disease Rating Scale; ADL, Activity of Daily Living; IADL, Instrumental Activity of Daily Living.*

**Table 2:** **Medications taken by the patients during the study**.

PD groups	N.	Levodopa	Dopamine agonists			MAO-inhibitors
			*Pramipexole*	*Ropinirole*	*Rotigotine*	*Rasagiline*
**Placebo**
	*1*	**+**		**+**		**+**
	*2*	**+**		**+**		
	*3*	**+**		**+**		
	*4*	**+**		**+**		
	*5*	**+**	**+**			**+**
	*6*	**+**		**+**		
	*7*	**+**				**+**
	*8*	**+**				
**Experimental**
	*1*	**+**				**+**
	*2*	**+**	**+**			**+**
	*3*	**+**	**+**			
	*4*	**+**				
	*5*	**+**			**+**	
	*6*	**+**	**+**			
	*7*	**+**	**+**			**+**

*PD, Parkinson’s disease; N., patient’s number; MAO-inhibitors, monoamine oxidase inhibitors.*

#### Neuropsychological Test Battery

Standardized tests were administered to PD patients to assess episodic memory [Immediate and Delayed Recall of a 15-Word List (Carlesimo et al., 1996); Prose Recall (Carlesimo et al., 2002); Immediate and delayed reproduction of the Rey’s Figure (Carlesimo et al., 2002)], attention and short-term memory [Digit Span and Corsi Block Tapping test Forward and Backward (Monaco et al., 2013); the Trail Making Test -Part A (Giovagnoli et al., 1996)], executive functions [Phonological Word Fluency (Carlesimo et al., 1996); Modified Card Sorting Test (MCST; Nocentini et al., 2002); Raven’s Colored Progressive Matrices (Carlesimo et al., 1996); the Trail Making Test -Part B (Giovagnoli et al., 1996)], language [Objects and Verbs Naming subtests from the Neuropsychological Examination of Aphasia (Capasso and Miceli, 2001)], visual-spatial functions [Copy of Drawings and Copy of Drawings with Landmarks (Carlesimo et al., 1996); Copy of the Rey’s Figure (Carlesimo et al., 2002)].

### Study Design and Procedure

In the experimental group, a 1-month 12-sessions treatment (three sessions weekly) that focused on the training of shifting abilities was administered. In each session, lasting 45 min, paper and pencil exercises involving different stimuli (e.g., letters, numbers, shapes) were proposed. The exercises were modeled on existing paradigms shown to be sensitive to frontal-striatal activity (MacDonald and Monchi, 2011). Exercises required subject to alternatively select between stimuli belonging to different semantic categories or between stimuli with different visual and spatial features.

Exercises were grouped in four modules, each requiring three sessions to be administered with increasing levels of difficulty (i.e., increasing the number of stimuli and reducing the time to complete the exercise). Subjects included in the study were asked to alternately select between stimuli with different visual and spatial features or according to their belonging to different semantic categories. For example, they had to alternately indicate figures representing living or non-living objects on a sheet of paper, join numbers with the corresponding letters (i.e., as in the Trail Making Test -Part B) or select stimuli on an arrow that were alternately close to or far from a target letter. The experimental protocol started with a basal module, followed by subsequent modules that were consecutively proposed.

In case the subjects did not reach the required level of accuracy on a module (80%), this was administered again. In fact, all patients reached the criteria established for all sections.

In the placebo group, a treatment with the same set characteristics as those of experimental one was administered (i.e., frequency, duration of each session and of the whole treatment). In this case, however, subjects were administered simple cognitive exercises for sustained attention and language abilities (dictation exercises and reordering of sentences sequences) that did not vary for difficulty degree across sessions, and respiratory exercises. For example, patients were read a text by the examiner that they had to write on a paper and were given syntactically incorrect sentences they had to reorder. In particular, for this group, half of each session was dedicated to cognitive activity and half to respiratory exercises. The examiners (both for behavioral and biochemical tests) were blinded to the arm the subject was assigned to.

### Zoo Map Test

In order to evaluate the effect of the shifting training on cognitive functions, we recorded the performance scores on the ZMT, a task mainly devoted to measure planning abilities included in the Behavioral Assessment of the Dysexecutive Syndrome battery (Wilson et al., 1998). The ZMT is composed by two consecutive trials in which the subject is required to visit six out of 12 locations on a zoo map, according to specified rules. In the first trial planning abilities are stressed by the fact that no instructions on the possible sequence is given. In the second trial, the difficulty of the test is reduced by providing instructions on the locations sequence. In this way a direct comparison between performances on the first vs. second trial allows the evaluation of planning functioning. In order to evaluate performance, execution time, and accuracy (range = 0–8 for each trial; this score takes into account the effects of errors made by the subject) are registered for both trials. The test was administered twice to all PD patients, before beginning the treatment (T0) and within 1 week from the end of treatment (T1). During the test, the patients were under their regular dopamine treatment. The test was given at the same time of the day at T0 at T1 to reduce possible confounding effects of dopamine therapy between the two sessions.

### Blood Sampling

Blood samples were taken between 8 and 10 p.m. at the beginning (T0) and within 1 week from the end of treatment (T1). Venous blood was collected into sampling tubes and centrifuged at 2000 × *g* for 20 min. Serum was then aliquoted and stored at –80°C until analysis.

### Determination of BDNF Content

Brain-derived neurotrophic factor (R&D Systems, USA; cat. N° DY248) was detected in sandwich ELISA according to the instructions of manufacturers. This sandwich ELISA is set in order to measure natural and recombinant human mature BDNF in serum and plasma. All assays were performed on F-bottom 96-well plates (Nunc, Wiesbaden, Germany). Tertiary antibodies were conjugated to horseradish peroxidase. Wells were developed with tetramethylbenzidine and measured at 450/570 nm. BDNF content was quantified against a standard curve calibrated with known amounts of protein. The detection limit for BDNF was 15 pg/ml. Measurements were performed in duplicate and values are expressed as ng/ml. Cross-reactivity to other related trophic factors (NGF, NT-3; NT-4; TGFβ, TGFα) was less than 3%.

### Statistical Analyses

In order to examine the effect of treatment on PD patients’ performance on cognitive test, two mixed ANOVAs were performed considering as dependent variable the accuracy (that includes the errors made by the participant) and response times, respectively. In the case of the ZMT the Treatment (experimental vs. placebo) was the between factor and Time of Assessment (T0 vs. T1) was the within factor. In the case of the ZMT the within factor Trial (Trial 1 vs. Trial 2) was added.

The same analyses were executed to investigate BDNF changes as a function of the cognitive training. In this case, with Treatment (experimental vs. placebo) as between factor and Time of Assessment (T0 vs. T1) as within factor. In all cases, LSD test was applied to qualify the statistical significance of main effects and interactions.

To examine the relationship between subjects’ performance changes between T0 vs. T1 changes on cognitive test and BDNF levels we executed Pearson’s r correlations analyses were execute on the PD group as a whole and separately for the two PD subgroups.

## Results

### Zoo Map Test

The results of ZMT are reported in **Table 3**.

**Table 3:** **Cognitive performances of Parkinson’s disease patients at the beginning (T0) and at the end (T1) of the treatment**.

Zoo Map Test	Time		Statistics		
	T0	T1	Cohen’ s *d*	*p*-value
**Trial 1**
Treatment	2.8 (2.6)	5.2 (2.3)	0.98	0.05
Placebo	2.4 (3.4)	2.5 (2.8)	0.03	>0.60
**Trial 2**
Treatment	8.0 (0)	6.3 (2.5)	1.30	>0.10
Placebo	6.0 (2.3)	6.9 (1.7)	0.45	>0.30

*Data are expressed as mean ± standard deviation (SD).*

#### Accuracy

There was a significant effect of Trial [*F*(1,13) = 53.0; *p* < 0.001] documenting that subjects were more accurate in Trial 2 (mean = 6.8; SD = 1.6) than in Trial 1 (mean = 3.2; SD = 2.8), and a Treatment*Time of Assessment*Trial interaction [*F*(2,13) = 5.29; *p* < 0.05]. *Post hoc* tests showed that subjects who underwent to experimental treatment significantly improved their performance passing from T0 to T1 in the Trial 1 (T0: mean = 2.8; SD = 2.6; T1: mean = 5.2; SD = 2.3; *p* = 0.05; Cohen’s *d* = 0.98), but not in trial 2 (T0: mean = 8.0; SD = 0; T1: mean = 6.3; SD = 2.5; *p* > 0.10; Cohen’s *d* = 1.30), while performance of subjects in the placebo group did not significantly change on both Trial 1 (T0: mean = 2.4; SD = 3.4; T1: mean = 2.5; SD = 2.8; *p* > 0.60; Cohen’s *d* = 0.03) and Trial 2 (T0: mean = 6.0; SD = 2.3; T1: mean = 6.9; SD = 1.7; *p* > 0.30; Cohen’s *d* = 0.45).

#### Response Times

There was a main effect of Trial [*F*(1,13) = 49.3; *p* < 0.001], documenting that all subjects were faster in performing Trial 2 (mean = 144.7; SD = 75.0) compared to Trial 1 (mean = 345.7; SD = 146.6); the effect of Time of Assessment only approached the statistical significance [*F*(1,13) = 4.14; *p* = 0.065]. None of the interactions involving the Treatment factor reached the level of statistical significance.

### BDNF Serum Levels

**FIGURE 1:**
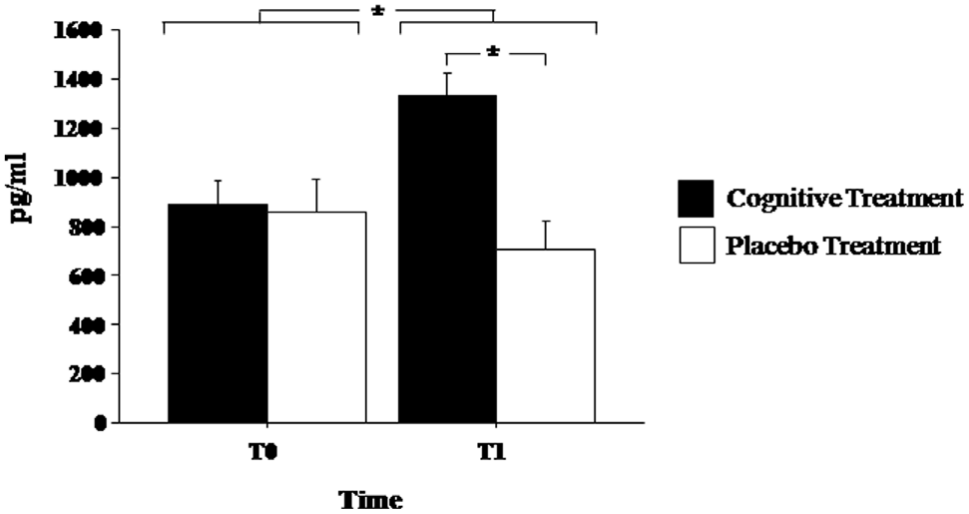
**Brain-derived neurotrophic factor (BDNF) serum levels in Parkinson’s disease patients before (T0) and after (T1) the cognitive rehabilitation protocol and placebo treatment.** Data are the mean ± SEM. Values are expressed in pg/ml. Asterisk (*) indicates significant difference between the groups. *p < 0.05.

Brain-derived neurotrophic factor serum levels before and at the end of experimental treatments are shown in **Figure 1**. Mixed ANOVA showed a significant effect of the main factor Treatment [*F*(1,13)= 8.272; *p*< 0.05] and of the Time of Assessment*Group interaction [*F*(1,13) = 6.883; *p* < 0.05]. *Post hoc* analyses showed that at the end of the treatment (T1) BDNF serum levels significantly increased in patients of the experimental group (*p* < 0.05) but not in the placebo group (*p* > 0.30). Moreover, at T1, BDNF levels were significantly elevated in the experimental group as compared to the placebo group (*p* < 0.01; **Figure 1**).

### Correlation between Cognitive Performance Changes and Changes on BDNF Levels

**FIGURE 2:**
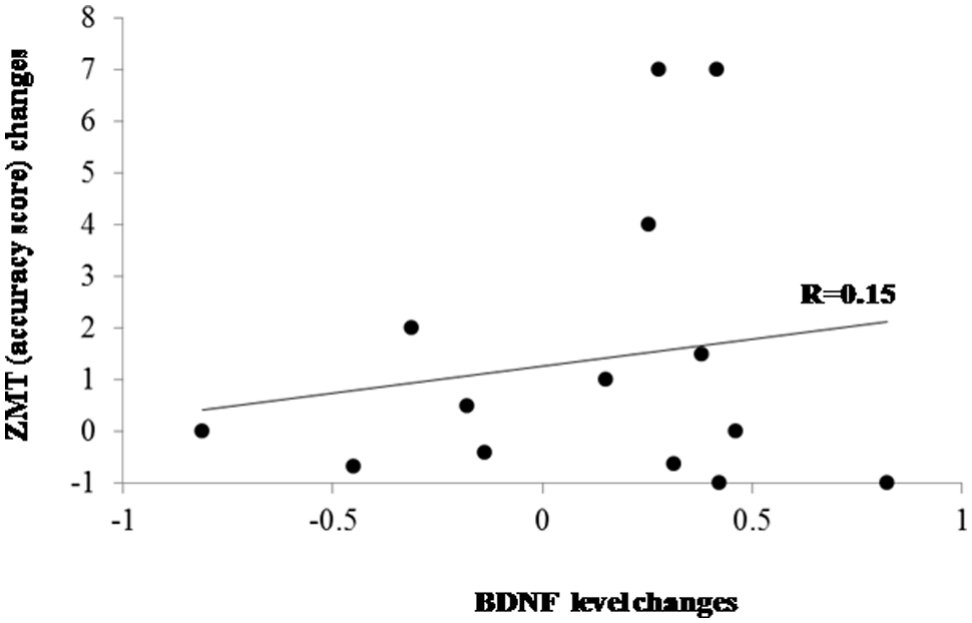
**Scatter plot evidencing the relationship between BDNF and cognitive changes after treatments in the whole PD group.** ZMT = Zoo Map Test.

Cognitive performance changes (in terms of accuracy) and BDNF changes passing from T0 to T1 were computed as percentage of improvement/worsening according to the following formula: (T1-T0)/T0. Correlation analyses did not show significant association between BDNF changes and changes on ZMT both in the PD group as a whole (*r* = 0.15; *p* > 0.60; **Figure 2**) and separately in the PD patients who underwent shifting training (*r* = 0.05) and in patients belonging to the placebo group (*r* = –0.06). The analysis of the difference between the r values of the two PD subgroups did not evidence significant effect (*z* < 0.01). We executed a further correlation between BDNF changes and response times changes on the ZMT trial 1 passing from T0 to T1 that, also in this case, did not evidence significant effects (*r* = –0.16; *p* > 0.10).

## Discussion

This study was performed to investigate whether a cognitive rehabilitation protocol focused on the training of shifting abilities was able to alter BDNF serum levels in PD patients affected by mild cognitive impairment. The results showed that PD patients undergoing the cognitive rehabilitation protocol, besides showing improved cognitive performance as measured with the ZMT also displayed increased serum levels of BDNF as compared to the placebo group.

To the best of our knowledge, this is the first study showing that cognitive rehabilitation in PD, besides having positive effects on cognitive functions, may also induce an increase in BDNF serum levels. The mechanism of action of cognitive rehabilitation has been investigated in MRI studies in patients with PD (Nombela et al., 2011). It was found that cognitive training improved the cognitive performance of the trained PD patients, with a cortical activation patterns comparable to those observed in controls. The neuronal circuits hypothesized are those of dorsolateral prefrontal cortex and basal ganglia, two regions strongly affected by dopamine depletion in PD, as well as the fronto-parietal circuitry.

The training here implemented was specifically structured to potentiate set-shifting, that is the ability to flexibly access to different mental representations/processes and responses according to the environmental demands. Indeed, cognitive flexibility is reported to be early impaired in PD patients, likely as a result of an imbalance of dopamine activity within key regions of frontal-striatal networks (i.e., caudate nucleus and prefrontal cortex; Cools and D’Esposito, 2011). This evidence makes cognitive flexibility an interesting target to investigate, in PD, both the effect of cognitive trainings and the possible neurobiological modifications related to BDNF activity. As a matter of fact, in the basal ganglia BDNF supports the survival and function of the dopaminergic neurons and for these reasons its role in PD pathogenesis and treatment of motor diseases has been widely investigated (He et al., 2013). Additionally, BDNF regulates synaptic plasticity and plays a critical role in maintaining normal prefrontal cortex function (Savitz et al., 2006; Woo and Lu, 2006), leading to the idea that BDNF is involved in the regulation of working memory and behavioral processes (Galloway et al., 2008; Sakata et al., 2013). Supporting this notion, previous reports from BDNF heterozygous knockouts and knockdowns revealed impairments in cognitive functions (Monteggia et al., 2004). Some authors reported that water maze performance is perturbed in forebrain-deleted BDNF mice (Gorski et al., 2003) and in mice with hippocampal virally induced BDNF ablation (Heldt et al., 2007), whereas transgenic overexpression of BDNF in the cerebral cortex and hippocampus facilitates performance (Koponen et al., 2004). In humans, molecular studies have evidenced that a functional polymorphism (Val66Met) in BDNF gene can influence human executive functions in healthy subjects (Alfimova et al., 2012) and in patients affected by mental illness (Lu et al., 2012; Tükel et al., 2012).

Other studies have shown that brain stimulation, in form of environmental or cognitive enrichment, may modulate BDNF in the brain. In animal models, environmental enrichment has been shown to increase BDNF levels in the hippocampus (Ramírez-Rodríguez et al., 2014), as well as other brain areas (Angelucci et al., 2009). Moreover, infusion of BDNF directly into the basal ganglia (nucleus accumbens) restored cognition, synaptic plasticity, and cell signaling in cognitively impaired aged rats (Li et al., 2012). In human subjects, it has been shown that BDNF increases after physical exercise (Schmolesky et al., 2013; Vaughan et al., 2014), and this increase may correlate to improvement of cognitive functions in pathological conditions such as stroke (El-Tamawy et al., 2014) and depression (Oral et al., 2012). Moreover, other studies have shown that BDNF serum levels in PD patients might change in relation to other types of rehabilitations such as intensive motor training and these effects are associated to motor improvements (Frazzitta et al., 2014). These results parallel those obtained in animal models where the increase in striatal BDNF has been associated to the neuroprotective effects of exercise training (Tuon et al., 2012; Real et al., 2013). These effects in animal models are not limited to PD but also to other neuropathological conditions such as stroke (Mang et al., 2013), Alzheimer’s (Dao et al., 2013) and Huntington’s (Pang et al., 2006) diseases, depression (Pereira et al., 2013) and spinal cord injury (Macias et al., 2009).

Altogether these data suggest that BDNF may have a dual role in the dopaminergic system. It is a protective agent of the nigrostriatal pathway with its survival action and can modulate cognitive processes by regulating synaptic plasticity in the hippocampal and cortical pathway. This dual role is of special relevance to PD. Several studies have shown an association between motor dysfunction and cognitive performance in PD patients. Specifically, among all the PD symptoms, bradykinesia has been found to be associated with poor performance in tests measuring mental flexibility and working memory (Domellöf et al., 2011). Bradykinesia is considered an hallmark of nigrostriatal lesion in PD (Vingerhoets et al., 1997). However, these findings suggest that loss of dopaminergic neurons may also cause non-motor symptoms. Supporting this hypothesis, it has been shown that both bradykinesia and aspects of cognition involving mental flexibility(Lewis et al., 2005) and working memory may benefit from intake of dopaminergic drugs (Lewis et al., 2003).

Brain-derived neurotrophic factor signaling, via its Tropomyosin related kinase B receptor tyrosine kinase, is important for the survival of nigrostriatal dopaminergic neurons (Baquet et al., 2004; Baydyuk et al., 2011). Thus, absence or reduced support of BDNF in dopaminergic nigrostriatal pathway may cause either reduced survival and/or malfunction. Interestingly, postmortem studies showed that BDNF mRNA is reduced in the substantia nigra (pars compacta) of PD patients as compared healthy subjects (Howells et al., 2000). Also, serum levels of BDNF are directly correlated with the amount of striatal dopamine transporter binding (Ziebell et al., 2012) and the severity of motor symptoms in PD (Scalzo et al., 2010). On the other hand, studies on BDNF gene expression have evidenced that this neurotrophin may exert its effect on cognitive functions (such as long-term memory and executive functioning) by regulating synaptic transmission in hippocampal and prefrontal regions (Savitz et al., 2006). These data indicate that BDNF may, at least in part, mediate the effect of cognitive rehabilitation in PD patients. However, given its dual role on the nigrostriatal and cortical pathways, the mechanism by which BDNF influences executive function may be wider and not limited to this specific task. Other cognitive functions, beside those investigated in the present study, may be involved and BDNF effect may be not limited to dopamine, but also to other neurotransmitters (i.e., glutamate) as recently demonstrated in mice (D’Amore et al., 2013). The fact that in our cohort of PD patients BDNF levels do not correlate with changes in executive function provides evidence for this hypothesis.

Limits of the study are represented by the relatively small sample size that could have affected the power of statistical analyses. For instance, low sample size could have prevented us from finding significant correlations between BDNF and cognitive performance changes. Thus, our data should be regarded as preliminary observations and need to be confirmed in larger cohorts of PD patients. Furthermore, it should be noted that the effects of the shifting training were not compared with those of training focused on other executive sub-components (e.g., updating or inhibition). Therefore, we cannot exclude the possibility that the effect observed in the PD group after cognitive training was due to a general improvement of executive/attentional functioning, rather than merely shifting abilities, also considering that the shifting training has a higher level of attention-demanding when compared to the placebo training.

Nonetheless, given that the placebo and the experimental groups were strictly comparable in terms of clinical and cognitive symptoms as well as dopamine medication, this pilot study can provide valuable information on the neurobiological correlates of cognitive rehabilitation in PD as well as in other neurodegenerative diseases (Vinogradov et al., 2009).

In conclusion, this pilot study showed that a cognitive rehabilitation program focused on the training of executive functioning improves cognitive functions and increases BDNF serum levels in PD patients with mild cognitive impairment. Additional studies with larger samples and/or other methodologies are needed to determine if BDNF acts directly in neural networks associated with executive functioning or indirectly via trophic influences on other neurotransmitters such as glutamate or dopamine.

## Author Contributions

FA gave substantial contributions to the conception of the work; to the acquisition, analysis, and interpretation of data for the work; Drafting the work or revising it critically for important intellectual content; Final approval of the version to be published; Agreement to be accountable for all aspects of the work in ensuring that questions related to the accuracy or integrity of any part of the work are appropriately investigated and resolved.

AP gave substantial contributions to the conception or design of the work; and the acquisition, analysis, or interpretation of data for the work; Drafting the work or revising it critically for important intellectual content; Final approval of the version to be published; Agreement to be accountable for all aspects of the work in ensuring that questions related to the accuracy or integrity of any part of the work are appropriately investigated and resolved.

GC gave substantial contributions to the conception or design of the work; Drafting the work or revising it critically for important intellectual content; Final approval of the version to be published; Agreement to be accountable for all aspects of the work in ensuring that questions related to the accuracy or integrity of any part of the work are appropriately investigated and resolved.

FS gave substantial contributions to the acquisition of data for the work; Drafting the work or revising it critically for important intellectual content; Final approval of the version to be published; Agreement to be accountable for all aspects of the work in ensuring that questions related to the accuracy or integrity of any part of the work are appropriately investigated and resolved.

SZ gave substantial contributions to the acquisition of data for the work; Drafting the work or revising it critically for important intellectual content; Final approval of the version to be published; Agreement to be accountable for all aspects of the work in ensuring that questions related to the accuracy or integrity of any part of the work are appropriately investigated and resolved.

FB gave substantial contributions to the acquisition of data for the work; Drafting the work or revising it critically for important intellectual content; Final approval of the version to be published; Agreement to be accountable for all aspects of the work in ensuring that questions related to the accuracy or integrity of any part of the work are appropriately investigated and resolved.

JS gave substantial contributions to the acquisition of data for the work; Drafting the work or revising it critically for important intellectual content; Final approval of the version to be published; Agreement to be accountable for all aspects of the work in ensuring that questions related to the accuracy or integrity of any part of the work are appropriately investigated and resolved.

CC gave substantial contributions to the conception or design of the work; Drafting the work or revising it critically for important intellectual content; Final approval of the version to be published; Agreement to be accountable for all aspects of the work in ensuring that questions related to the accuracy or integrity of any part of the work are appropriately investigated and resolved.

AC gave substantial contributions to the conception or design of the work; or the acquisition, analysis, or interpretation of data for the work; Drafting the work or revising it critically for important intellectual content; Final approval of the version to be published; Agreement to be accountable for all aspects of the work in ensuring that questions related to the accuracy or integrity of any part of the work are appropriately investigated and resolved.

## Conflict of Interest Statement

The authors declare that the research was conducted in the absence of any commercial or financial relationships that could be construed as a potential conflict of interest.
